# Lack of serological and molecular evidences of Zika virus circulation in non-human primates in three states from Brazil

**DOI:** 10.1590/0074-02760220012

**Published:** 2022-09-05

**Authors:** Amanda Haisi, Stacy Wu, Nathalia Zini, Maria Luana Cristiny Rodrigues da Silva, Camila Dantas Malossi, Zalmir Silvino Cubas, Patrícia Hoerner Cubas, Rodrigo Hidalgo Friciello Teixeira, Mônica Shinneider de Sousa, Ricardo Barbosa Lucena, Walfrido Kühl Svoboda, Silvia Cristina Osaki, Mauricio Lacerda Nogueira, Leila Sabrina Ullmann, João Pessoa Araújo

**Affiliations:** 1 Universidade Estadual Paulista Júlio de Mesquita Filho, Instituto de Biotecnologia, Botucatu, SP, Brasil; 2 Universidade Federal do Paraná, Departamento de Ciências Veterinárias, Palotina, PR, Brasil; 3 Escola de Medicina de São José do Rio Preto, Laboratório de Pesquisas em Virologia, São José do Rio Preto, SP, Brasil; 4 Universidade Federal de Campina Grande, Patos, PB, Brasil; 5 Refúgio Biológico Bela Vista, Itaipu Binacional, Foz do Iguaçu, PR, Brasil; 6 Zoológico Bosque do Guarani, Foz do Iguaçu, PR, Brasil; 7 Zoológico Municipal Quinzinho de Barros, Sorocaba, SP, Brasil; 8 Universidade de Sorocaba, Sorocaba, SP, Brasil; 9 Universidade Federal da Paraíba, Departamento de Ciências Veterinárias, Areia, PB, Brasil; 10 Universidade Federal da Integração Latino-Americana, Foz do Iguaçu, PR, Brasil; 11 University of Texas Medical Branch, Department of Pathology, Galveston, Texas, USA; 12 Universidade Federal de Mato Grosso do Sul, Faculdade de Medicina Veterinária e Zootecnia, Campo Grande, MS, Brasil

**Keywords:** arbovirus, non-human primates, public health, sylvatic cycle

## Abstract

**BACKGROUND:**

Zika virus (ZIKV) was discovered in 1947 with the virus isolation from Rhesus monkey (*Macaca mulatta*) in Uganda forest, Africa. Old World Primates are involved in a sylvatic cycle of maintenance of this arbovirus, however a limited knowledge about the role of New World primates in ZIKV transmission cycles has been established.

**OBJECTIVE:**

This work aimed to investigate the presence of enzootic circulation of ZIKV in New World Primates from three Brazilian states: São Paulo, Paraíba, and Paraná.

**METHODS:**

We analyzed 100 non-human primate samples collected in 2018 and 2020 from free-ranging and captive environments from São Paulo (six municipalities belonging to Sorocaba region), Paraíba (João Pessoa municipality), and Paraná (Foz do Iguaçu municipality) using reverse transcriptase quantitative polymerase reaction (RT-qPCR) assays, indirect enzyme-linked immunosorbent assay (ELISA), and plaque reduction neutralization test (PRNT).

**FINDINGS:**

All samples (n = 141) tested negative for the presence of ZIKV genome from tissue and blood samples. In addition, all sera (n = 58) from Foz do Iguaçu’ non-human primates (NHPs) were negative in serological assays.

**MAIN CONCLUSION:**

No evidence of ZIKV circulation (molecular and serological) was found in neotropical primates. In addition, the absence of antibodies against ZIKV suggests the absence of previous viral exposure of NHPs from Foz do Iguaçu-PR.

Approximately 75% of emerging pathogens in humans are shared with animal reservoirs.^1^ Specifically, non-human primates (NHP) are the closest phylogenetically animals to humans and contribute with 20% of major human diseases.^2^ In addition, these mammals can act as natural sentinels for surveillance of emerging diseases, including arboviruses, mainly due to the presence of these animals in urban and peri-urban areas and also the proximity of some species to human activities which favors the events of spillback and spillover of these agents.^3^ ^,^ ^4^ ^,^ ^5^

Old World primates (OWP) are involved in the maintenance of the enzootic cycle of arboviruses of particular importance for public health, such as Zika virus (ZIKV) and dengue virus (DENV) in Africa, and yellow fever virus (YFV) in Asia.^6^ In Brazil, the evidence of natural susceptibility of New World primates (NWP) to ZIKV infection was first described in *Callithrix jacchus* and *Sapajus libidinosus* from Northeast region.^7^ Similarly, the same genera *Callithrix* sp. and *Sapajus* sp. were positive to ZIKV in the Southeast region of the country and epidemiologically associated with positive *Aedes aegypti* in the same area, suggesting a ZIKV spillback event by transmission between urban vectors to NHP.^3^ After that, only limited evidence of infection was reported, all involving low titers of neutralizing antibodies^8^ or associated with cross-reactivity with other flaviviruses in plaque reduction neutralization test (PRNT).^9^

The establishment of the ZIKV sylvatic cycle involving neotropical primates in South America, mainly in Brazil, is supported by several factors, including the presence of susceptible vectors and abundant diversity of non-human primates species potentially capable of acting as hosts for the sylvatic ZIKV cycle.^4^ This scenario, as occurred with the spillback from the urban to the YVF sylvatic cycle in South Americas,^10^ ^,^ ^11^ would provide a new dynamic transmission with immensurable negative impacts for both biodiversity and public health. Therefore, the main objective of the present study was to obtain evidence of the enzootic ZIKV circulation in free-ranging and captive non-human primates from Brazil.

## MATERIALS AND METHODS

*Ethics* - The Ethics Committee approved the present study in the Animal Experimentation (protocol CEUA/UNESP: 0206/2019), and the Brazilian Ministry of Environment (SISBIO: 67891/2019).

*Study areas* - The present study was carried out in three different states: São Paulo, Paraíba and Paraná states (Figure). Tissue samples were collected from dead or recently dead animals from 2018 to 2019 from metropolitan region of Sorocaba (São Paulo State) and João Pessoa municipality (Paraíba State). Free-ranging animals from João Pessoa were collected in the Botanical Garden Benjamim Maranhão, and one captive animal located in Zoobotanical park Arruda Câmara (BICA) was also sampled, both located in the urban area. The collection of free-ranging NHP samples from São Paulo State was performed during the yellow fever (YF) epizooty in six municipalities belonging to the metropolitan region of Sorocaba: Sarapuí, Tapiraí, Itu, Aroçaiaba da Serra and Capela do Alto.

**Figure:**
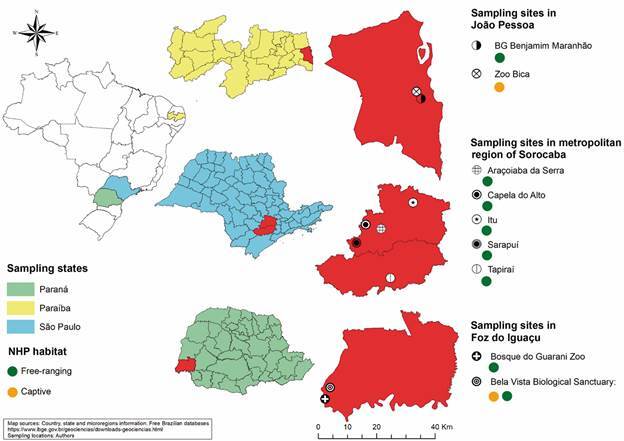
Sampling sites of non-human primates and the habitat of origin. On the left is the map of Brazil, highlighting the three Brazilian states, Paraíba (in yellow), São Paulo (in blue) and Paraná (in green). At greater magnification, the collection sites within the João Pessoa municipality, metropolitan region of Sorocaba and Foz do Iguaçu municipality. The habitat of non-human primates are represented by colored circles (green for free-ranging; yellow for captive). The image was designed using ArcGIS 10.8 software (https://www.esri.com).

In Paraná State, the NHP collection was carried out in Foz do Iguaçu (25º 30’ 58”; S 54º 35’ 07” W), the municipality on the Triple Border with Argentina and Paraguay with approximately 250,000 habitant,^12^ from December 2019 to March 2020, characterized as an active surveillance study collecting captive and free-ranging neotropical primates. The captive animals are from Zoological Bosque do Guarani, localized in the urban area and the Roberto Ribas Lange Zoological situated into the Refúgio Biológico Bela Vista (RBV) - ITAIPU in the rural area. Free-ranging black capuchins (*Sapajus nigritus*) were captured into the Permanent Protection Area of Itaipu Binacional (PPA-IB) that comprises the RBV area.

*Biological Samples* - Tissue samples were collected from 33 free-ranging NHP found dead in the metropolitan region of Sorocaba (n = 12) and João Pessoa municipality (n = 21), including, in the last location, one captive NHP from BICA that died during the same period of collection and was also included in this study.

All NHP carcasses were subjected to necropsy performed by local authorities on health services and all tissue samples (n = 75) (brain, heart, kidneys, liver, lungs and spleen) from Sorocaba region (n = 38) and João Pessoa municipality (n = 37) were submitted to the Biotechnology Institute, UNESP, to ZIKV diagnose. Samples from João Pessoa municipalty were stored in RNA*later* solution and all tissue samples were stored at -80ºC until use.

A total of sixty-six NHP from Foz do Iguaçu were sampled (n = 38 captive and n = 28 free-ranging). The free-ranging animals were captured at the APP-IB using Tomahawk traps. In both situations, captive and free-ranging NHP were anesthetized with an association of ketamine hydrochlorine (10 mg/kg), xylazine (0.5mg/kg) and midazolam maleate (0.2 mg/kg). Blood (n = 66) and serum (n = 58) samples were collected using Vacutainer^*®*^ (BD) in EDTA - anticoagulated tubes and in gel separator tubes for molecular and serological assays, respectively. All samples were stored at -80ºC until use. In addition, all animals were physically examined, marked with microchips and returned to the origin place (captive or free-range) after complete recovery from the anesthetic’s effects.

*Polymerase chain reaction (PCR) assays* - RNA was extracted from frozen tissues (n = 75) using a commercial kit (RNeasy, Qiagen, Hilden, Germany) with 1.4 mm and 3.0 mm zirconium beads (Locus, Cotia, SP) in a speed blender (Next advance, Troy NY EUA), following the manufacturer’s instructions. For blood samples (n = 66), RNA was extracted from 200 µL using a commercial ReliaPrep Viral TNA MiniPrep TNA kit (Promega, Madison, USA), according to the manufacture’s recommendations. In both, RNA was eluted in 50 microliters.

All samples were analyzed for the presence of ZIKV by a TaqMan^®^ reverse transcriptase quantitative polymerase reaction (RT-qPCR), as previously described, with primers targeting the envelope gene.^13^ The qPCR was performed using the KiCqStart One-Step Probe RT-qPCR ReadyMix™ 2X master mix (Merck, Germany), according to the manufacturer’s instructions and using the AriaMX real time PCR System (Agilent, Santa Clara, CA, EUA). The viral strains used as positive controls were the ZIKV^BR^ (Bioscience Institute, USP, Brazil).

*Serological assays* - Serum samples (n = 58) were tested to determine the specific neutralization antibodies titers in a PRNT with a modified protocol previously described.^14^ The PRNT was performed in 24-well plates (Costar^®^, Corning Incorporated, NY, USA) with Vero E6 cells/well-kept in Eagle’s Minimum Essential Medium (MEM, Cultilab, Brazil), using a fixed ZIKV^BR^ (Instituto Evandro Chagas) virus inoculum (~50 PFU) against varying serum dilutions (1:5 to 1:80). The plates were overlaid with a semi-solid medium [MEM 1×, 1% fetal bovine serum (FBS), 1.5% carboxymethylcellulose] and incubated at 37ºC in 5% CO_2_ for four days. After that, the cells’ monolayer was fixed with a 10% formalin solution and 2% crystal violet solution. Neutralizing antibody titers were expressed by 80% of plaque reduction (PRNT_80%_). Because of the low specificity of anti-flavivirus antibodies, serum samples that presented PRNT_80%_ titers for ZIKV ≤ 5, in either monotypic or heterotypic reactions, were considered seronegative.

The indirect enzyme-linked immunosorbent assay (ELISA) was conducted in all serum samples to detect specific IgG titers using a commercial test. Anti-ZIKV ELISA (IgG) (Euroimmun, Lübeeck, Germany) was performed according to the manufacturer’s recommendations. Samples with an immune status score > 1.1 were considered IgG positive, between ≥ 0.8 and 1.1 undetermined, and ≤ 0.8 were negative for ZIKV. The ELISA results were calculated from the ratio between the mean of the optical density (OD) of the calibrators by the OD of the samples tested.

## RESULTS

*Non-human primate samples* - A total of 141 samples were collected from 100 NHP (Table) and analyzed for the presence of ZIKV infection by RT-qPCR. Detailed information for each specimen (species, sex, samples tested, site of sampling) was clusterized into animals from active surveillance from Foz do Iguaçu-PR [Supplementary data (Table I)] and passive surveillance with collection of tissue samples from animals found dead from João Pessoa-PB and the metropolitan region of Sorocaba-SP [Supplementary data (Table II)].

**TABLE:** Diversity of neotropical primates collected during 2018 to 2020 in three Brazilian states: São Paulo, Paraíba and Paraná

Species	Situation	State	Sex			Total
			F	M	I	
Southern Brown Howler Monkey (*Alouatta clamitans*)	Free-ranging	São Paulo	1	3	0	4
Black-capped Capuchin (*Sapajus apella*)			0	1	2	3
Common marmoset (*Callithrix jacchus*)			3	1	0	4
Black-penicilled Marmoset (*Callithrix penicillata*)			0	1	0	1
Capuchin monkey (*Sapajus* sp.)	Free-ranging	Paraíba	0	1	0	1
Common marmoset (*Callithrix jacchus*)			13	7	0	20
Guianan squirrel monkey (*Saimiri sciureus*)	Captive from Zoobotanical Garden (Zoo Bica)		1	0	0	1
Black-horned Capuchin (*Sapajus nigritus*)	Free-ranging (Bela Vista Sanctuary)	Paraná	7	21	0	28
Northern Brown Howler Monkey (*Alouatta guariba*)	Captive (Roberto Ribas Lange Zoo)		3	3	0	6
Black-horned Capuchin (*Sapajus nigritus*)			3	5	0	8
Black Howler monkey (*Alouatta caraya*)	Captive (Bosque do Guarani Zoo)		1	0	0	1
Black-horned Capuchin (*Sapajus nigritus*)			9	6	0	15
Golden-headed Lion Tamarin (*Leontopithecus chrysomelas*)			1	2	0	3
Black-penicilled Marmoset (*Callithrix penicillata*)			4	1	0	5
Total			46	52	2	100

F: female; M: male; I: indeterminate or not informed.

*Molecular detection results* - All samples (n = 141) tested negative to the presence of ZIKV genome RNA, regardless of the collection site or epidemiological situation.

*Serological results* - A total of 58 serum samples from Foz do Iguaçu-PR were screened for neutralizing antibodies to ZIKV. The sera of eight animals were not available due to the small size of the animals, preventing the collection of ideal amounts of blood for molecular and serology tests.

All serum samples were considered negative for IgG against ZIKV by the commercial indirect ELISA. The OD values for ELISA are detailed in Supplementary data (Table III). Only one free-ranging NHP showed the presence of neutralization antibodies end titers ≤ 1:5 (dilution 1:5) in PRNT_80%_ and was considered negative to presence of neutralization antibodies for ZIKV. This unique sample was also tested in PRNT for DENV 1-4, YFV, Chikungunya virus (CHIKV)^15^ and also was considered negative.

## DISCUSSION

The role of neotropical primates in the epidemiology and transmission of arbovirus, such as ZIKV, is not fully clarified, however several reports demonstrate that NHP are susceptible to infection in South America.^3^ ^,^ ^7^ In this study, we analysed the presence of RNA from ZIKV in the metropolitan region of Sorocaba (São Paulo State), João Pessoa (Paraíba State), and Foz do Iguaçu (Paraná State), and the presence of antibodies for ZIKV in the last municipality.

Negative results from RT-qPCR in our study suggest that no active infection by ZIKV was present in these 100 neotropical primates (66 from Foz do Iguaçu; 22 from João Pessoa and 12 from São Paulo) collected and that they probably were not involved in an enzootic transmission in the studied sites, including the epidemiological period of YF outbreak in southeast Brazil (2018) and after the emergence of ZIKV in Brazil (2015-2016). Similar results were observed in NHP sampled in Rio de Janeiro and São Paulo during and before YF outbreak.^9^ Additionally, other studies had not found evidence of ZIKV infection in domestic and wild animals,^8^ ^,^ ^16^ including after and during the ZIKV circulation (2012-2016) in Paraíba State.^8^

Humans are considered the unique host involved in the urban ZIKV transmission cycle involving *Aedes* spp. in urban and peri-urban areas.^17^ *Ae. aegypti* is a common urban mosquito with a highly adapted to live in association in closes contact with humans in urbanized areas, and the mainly competent vector for ZIKV in Brazil.^17^ ^,^ ^18^ Despite the preference for human host, other available mammals may serve as a blood smear for these antropophilic mosquito, including non-human primates.^19^ ^,^ ^20^

In contrast with Terzian et al.,^3^ that demonstrated free-living marmosets and capuchin monkeys naturally infected with ZIKV in areas of intense ZIKV circulation, in this study, all free-ranging NHP from the metropolitan region of São Paulo were collected during 2018 and 2019, when 126 autochthonous cases were notified (in one year period 2018), out of these, just one case corresponding to Tapiraí municipality^21^ one of the five municipalities in this study, suggesting a low viral circulation when compared with the period of emergence of ZIKV (2016) with 3,857 autochthone cases notified in São Paulo State.^22^

A recently study in Foz do Iguaçu trapped 11,962 mosquitoes of *Ae. aegypti* in adult traps during 2017-2020 and the analysis of 221 pools tested (< 10 mosquitoes per pool) showed that 22 (75.9%) were positive for DENV and three were positive for ZIKV.^23^ Although Foz do Iguaçu presents the greatest number of autochthonous dengue cases among the cities of Paraná State, the notified cases of Zika (0.01%; 4/232) in one-year period (June 2019 and June 2020).^24^

A massive decline in human cases was observed in the period after the emergence of ZIKV (2016). These fact may suggest lower viral circulation of ZIKV in urban/periurban areas and consequently, lower opportunities for infected *Aedes* spp. to feed in neotropical primates, as observed in previous studies with African Green monkeys.^19^ In addition, the absence of ZIKV infection in free-ranging animals that possibly will have contact with sylvatic vectors may indicate the potential absence of primatophilic competent vectors in an enzootic cycle.^25^ Although we have not sampled mosquitoes, the refractory infection and low competence viral were observed in an experimental study with five wild neotropical mosquito species including *Haemagogus leucocelaenu*,^26^ considered one of the primary sylvatic YFV vectors in Brazil.^27^

Analysis of serological results requires careful evaluation especially when co-circulation of multiple arbovirus occur. Although we have observed a weak monotypic neutralization in a gold standard PRNT with a conservative limit of 80% neutralization to ZIKV without association of serologic detection for DENV, CHIKV and YFV, we cannot exclude there may be other active flaviviruses circulation in our study sites, such as Saint. Louis encephalitis virus (SLEV) and West Nile virus (WNV).^28^ In Argentina, country that makes the Triple Border with Foz do Iguaçu, the previous circulation of WNV, SLEV was reported in black howler (*Alouatta caraya*).^29^ This criteria may appear conservative, but the main objective is to prevent the introduction of false positives in the data.

During one year period (2019) in Paraíba State, 443 suspect cases were notified, approximately 10.75% more cases that 2018.^30^ Unfortunately, the only available samples of free-ranging neotropical primates from the northeast and southeast Brazil were tissue samples, the lack of serological data impossibilited to determinate the real absence of past exposure to the ZIKV. Therefore, further studies are encouraged, mainly due to the presence of neutralization antibodies in potential hosts,^8^ favorable climate to vectors proliferation and a great diversity of wild fauna that can serve as reservoir for ZIKV in Brazil.^4^

For all sites analyzed, the absence of ZIKV circulation during the collection of these animals does not exclude the importance of the constant monitoring by active surveillance with capture and sampling animals and passive surveillance by investigation of illness or death NHP in order to find evidence of enzootic ZIKV circulation in non-humans primates^4^ and to assess the impact on the biodiversity and humans.

In conclusion, these 100 neotropical primates from three Brazilian states had not participated in an enzootic cycle of maintenance of ZIKV. Even though there is no molecular and serological evidences of ZIKV infection in neotropical primates in these sampled sites, we emphasize the importance of monitoring these mammals as a surveillance tool, due to a possible establishment of a sylvatic cycle of maintenance, such as demonstrated for YVF in South Americas, and for the possibility of spillback event by transmission between urban vectors to NHP.
