# Supplementary material for: Lack of serological and molecular evidences of Zika virus circulation in non-human primates in three states from Brazil
Source: Mem Inst Oswaldo Cruz. 2022 Sep 5;117:e220012. doi: 10.1590/0074-02760220012 (PMC9444137; doi:10.1590/0074-02760220012)
Supplement: Supplementary file 1 [file 1678-8060-mioc-117-e220012-s.pdf]

TABLE I

Diversity of non-human primates (NHPs) samples from Foz do Iguaçu, Paraná State (2018-2019)

Species	Situation	Sex		Total	Samples	
		F	M		Blood	Serum
Black-horned Capuchin (*Sapajus nigritus*)	Free-ranging (APP-IB)	7	21	28	28	28
Northern Brown Howler Monkey (*Alouatta guariba*)	Captive	3	3	6	6	6
Black-horned Capuchin (*Sapajus nigritus*)	(Roberto Ribas Lange Zoo)	3	5	8	8	8
Black Howler monkey (*Alouatta caraya*)	Captive (Bosque do Guarani Zoo)	1	0	1	1	1
Black-horned Capuchin (*Sapajus nigritus*)		9	6	15	15	15
Golden-headed Lion Tamarin (*Leontopithecus chrysomelas*)		1	2	3	3	0
Black-penicilled Marmoset (*Callithrix penicillata*)		4	1	5	5	0
Total		28	38	66	66	58

F: female; M: male.

CC BY

TABLE II
Tissue samples of non-human primate (NHP) from São Paulo and Paraiba states (2018-2019)

State	Municipality	Species	Situation	Sex	Tissue samples						Total
					S	H	L	LG	K	B	
São Paulo	Sarapuí	Southern Brown Howler Monkey (*Alouatta clamitans*)	Free-ranging	M	-	2	2	-	-	-	4
	Tapiraí	Southern Brown Howler Monkey (*Alouatta clamitans*)	Free-ranging	F	-	-	1	-	-	2	3
	Sarapuí	Southern Brown Howler Monkey (*Alouatta clamitans*)	Free-ranging	M	-	2	1	2	-	-	5
	Sarapuí	Southern Brown Howler Monkey (*Alouatta clamitans*)	Free-ranging	M	-	-	2	-	-	-	2
	Itu	Black-capped Capuchin (*Sapajus apella*)	Free-ranging	M	-	2	2	-	-	-	4
	-	Black-capped Capuchin (*Sapajus apella*)	Free-ranging	I	-	-	1	-	-	-	1
	-	Black-capped Capuchin (*Sapajus apella*)	Free-ranging	I	-	-	1	-	-	-	1
	Capela do Alto	Common marmoset (*Callithrix jacchus*)	Free-ranging	F	-	-	2	-	1	-	3
	Araçoiaba da Serra	Common marmoset (*Callithrix jacchus*)	Free-ranging	F	2	2	2	1	-	-	7
	Capela do Alto	Common marmoset (*Callithrix jacchus*)	Free-ranging	F	-	1	-	1	-	-	2
	Capela do Alto	Common marmoset (*Callithrix jacchus*)	Free-ranging	F	-	1	-	1	2	-	4
	Itu	Black-penicilled Marmoset (*Callithrix penicillata*)	Free-ranging	M	-	1	1	-	-	-	2
Paraiba	João Pessoa	Capuchin monkey (*Sapajus* sp.)	Free-ranging	M	1	-	-	-	-	-	1
	João Pessoa	Common marmoset (*Callithrix jacchus*)	Free-ranging	F	-	-	1	-	1	1	3
	João Pessoa	Common marmoset (*Callithrix jacchus*)	Free-ranging	F	-	-	1	-	-	1	2
	João Pessoa	Common marmoset (*Callithrix jacchus*)	Free-ranging	F	-	-	-	-	-	1	1
	João Pessoa	Common marmoset (*Callithrix jacchus*)	Free-ranging	F	-	-	1	-	-	1	2
	João Pessoa	Common marmoset (*Callithrix jacchus*)	Free-ranging	F	-	-	1	-	-	1	2
	João Pessoa	Common marmoset (*Callithrix jacchus*)	Free-ranging	F	-	-	-	-	-	1	1
	João Pessoa	Common marmoset (*Callithrix jacchus*)	Free-ranging	F	-	-	-	-	-	1	1
	João Pessoa	Common marmoset (*Callithrix jacchus*)	Free-ranging	F	-	-	1	-	-	-	1
Paraiba	João Pessoa	Common marmoset (*Callithrix jacchus*)	Free-ranging	F	-	-	1	-	-	-	1
	João Pessoa	Common marmoset (*Callithrix jacchus*)	Free-ranging	F	-	-	1	-	-	-	1
	João Pessoa	Common marmoset (*Callithrix jacchus*)	Free-ranging	F	-	-	-	-	-	1	1
	João Pessoa	Common marmoset (*Callithrix jacchus*)	Free-ranging	F	1	-	1	1	-	1	4
	João Pessoa	Common marmoset (*Callithrix jacchus*)	Free-ranging	M	1	1	1	-	1	-	4
	João Pessoa	Common marmoset (*Callithrix jacchus*)	Free-ranging	M	1	-	1	1	1		4
	João Pessoa	Common marmoset (*Callithrix jacchus*)	Free-ranging	M	-	-	-	1	-	-	1
	João Pessoa	Common marmoset (*Callithrix jacchus*)	Free-ranging	M	-	-	-	-	1	-	1
	João Pessoa	Common marmoset (*Callithrix jacchus*)	Free-ranging	M	-	-	-	-	-	1	1
	João Pessoa	Common marmoset (*Callithrix jacchus*)	Free-ranging	M	-	-	1	-	-	-	1
	João Pessoa	Common marmoset (*Callithrix jacchus*)	Free-ranging	M	-	-	-	1	-	-	1
	João Pessoa	Guianan squirrel monkey (*Saimiri sciureus*)	Captive from Zoobotanical Garden	F	-	-	1	-	-	-	1
Total					6	12	27	10	7	13	75

F: female; M: male; I: indeterminate; S: spleen; H: heart; L: liver; LG: lung; K: kidney; B: brain.

TABLE III

Results of plaque reduction neutralization test (PRNT) 80% and indirect enzyme-linked immunosorbent assay (ELISA) for Zika virus (ZIKV) detection in 58 serum samples of non-human primate (NHP) from Foz do Iguaçu, Paraná State

Specie	Site of sampling	Habitat	Results		Imunologial conclusion
			PRNT 80%	ELISA OD*	
Sapajus nigritus	Roberto Ribas Lange Zoo	Captive	Negative	0.22	Negative
Sapajus nigritus	Roberto Ribas Lange Zoo	Captive	Negative	0.21	Negative
Sapajus nigritus	Roberto Ribas Lange Zoo	Captive	Negative	0.16	Negative
Sapajus nigritus	Roberto Ribas Lange Zoo	Captive	Negative	0.17	Negative
Sapajus nigritus	Roberto Ribas Lange Zoo	Captive	Negative	0.16	Negative
Sapajus nigritus	Roberto Ribas Lange Zoo	Captive	Negative	0.14	Negative
Sapajus nigritus	Roberto Ribas Lange Zoo	Captive	Negative	0.13	Negative
Sapajus nigritus	Roberto Ribas Lange Zoo	Captive	Negative	0.16	Negative
Sapajus nigritus	Bela Vista Sanctuary	Free-ranging	Negative	0.15	Negative
Sapajus nigritus	Bela Vista Sanctuary	Free-ranging	Negative	0.16	Negative
Sapajus nigritus	Bosque do Guarani Zoo	Captive	Negative	0.187	Negative
Sapajus nigritus	Bosque do Guarani Zoo	Captive	Negative	0.13	Negative
Sapajus nigritus	Bosque do Guarani Zoo	Captive	Negative	0.28	Negative
Sapajus nigritus	Bosque do Guarani Zoo	Captive	Negative	0.14	Negative
Sapajus nigritus	Bosque do Guarani Zoo	Captive	Negative	0.14	Negative
Sapajus nigritus	Bosque do Guarani Zoo	Captive	Negative	0.17	Negative
Sapajus nigritus	Bosque do Guarani Zoo	Captive	Negative	0.18	Negative
Sapajus nigritus	Bosque do Guarani Zoo	Captive	Negative	0.18	Negative
Sapajus nigritus	Bosque do Guarani Zoo	Captive	Negative	0.26	Negative
Sapajus nigritus	Bosque do Guarani Zoo	Captive	Negative	0.14	Negative
Sapajus nigritus	Bosque do Guarani Zoo	Captive	Negative	0.15	Negative
Sapajus nigritus	Bosque do Guarani Zoo	Captive	Negative	0.14	Negative
Sapajus nigritus	Bosque do Guarani Zoo	Captive	Negative	0.14	Negative
Sapajus nigritus	Bosque do Guarani Zoo	Captive	Negative	0.15	Negative
Sapajus nigritus	Bosque do Guarani Zoo	Captive	Negative	0.16	Negative
Sapajus nigritus	Bela Vista Sanctuary	Free-ranging	1:5	0.18	Negative
Sapajus nigritus	Bela Vista Sanctuary	Free-ranging	Negative	0.28	Negative
Sapajus nigritus	Bela Vista Sanctuary	Free-ranging	Negative	0.24	Negative
Sapajus nigritus	Bela Vista Sanctuary	Free-ranging	Negative	0.13	Negative
Sapajus nigritus	Bela Vista Sanctuary	Free-ranging	Negative	0.17	Negative
Sapajus nigritus	Bela Vista Sanctuary	Free-ranging	Negative	0.16	Negative
Sapajus nigritus	Bela Vista Sanctuary	Free-ranging	Negative	0.14	Negative
Sapajus nigritus	Bela Vista Sanctuary	Free-ranging	Negative	0.14	Negative
Sapajus nigritus	Bela Vista Sanctuary	Free-ranging	Negative	0.16	Negative
Sapajus nigritus	Bela Vista Sanctuary	Free-ranging	Negative	0.14	Negative
Sapajus nigritus	Bela Vista Sanctuary	Free-ranging	Negative	0.13	Negative
Sapajus nigritus	Bela Vista Sanctuary	Free-ranging	Negative	0.14	Negative
Sapajus nigritus	Bela Vista Sanctuary	Free-ranging	Negative	0.14	Negative
Sapajus nigritus	Bela Vista Sanctuary	Free-ranging	Negative	0.14	Negative
Sapajus nigritus	Bela Vista Sanctuary	Free-ranging	Negative	0.16	Negative
Sapajus nigritus	Bela Vista Sanctuary	Free-ranging	Negative	0.133	Negative

Specie	Site of sampling	Habitat	Results		Imunologial conclusion
			PRNT 80%	ELISA OD*	
Sapajus nigritus	Bela Vista Sanctuary	Free-ranging	Negative	0.13	Negative
Sapajus nigritus	Bela Vista Sanctuary	Free-ranging	Negative	0.14	Negative
Sapajus nigritus	Bela Vista Sanctuary	Free-ranging	Negative	0.22	Negative
Sapajus nigritus	Bela Vista Sanctuary	Free-ranging	Negative	0.15	Negative
Sapajus nigritus	Bela Vista Sanctuary	Free-ranging	Negative	0.19	Negative
Sapajus nigritus	Bela Vista Sanctuary	Free-ranging	Negative	0.17	Negative
Sapajus nigritus	Bela Vista Sanctuary	Free-ranging	Negative	0.24	Negative
Sapajus nigritus	Bela Vista Sanctuary	Free-ranging	Negative	0.13	Negative
Sapajus nigritus	Bela Vista Sanctuary	Free-ranging	Negative	0.14	Negative
Sapajus nigritus	Bela Vista Sanctuary	Free-ranging	Negative	0.14	Negative
Alouatta guariba	Roberto Ribas Lange Zoo	Captive	Negative	0.15	Negative
Alouatta guariba	Roberto Ribas Lange Zoo	Captive	Negative	0.13	Negative
Alouatta guariba	Roberto Ribas Lange Zoo	Captive	Negative	0.17	Negative
Alouatta guariba	Roberto Ribas Lange Zoo	Captive	Negative	0.14	Negative
Alouatta guariba	Roberto Ribas Lange Zoo	Captive	Negative	0.15	Negative
Alouatta guariba	Roberto Ribas Lange Zoo	Captive	Negative	0.13	Negative
Alouatta caraya	Bosque do Guarani Zoo	Captive	Negative	0.15	Negative

*The ELISA results were calculated from the ratio between the mean of the optical density (OD) of the calibrators by the OD of the samples tested.
